# A Systematic Review of Gastrointestinal and Respiratory Pathogen Detection in Wastewater in Africa, with Focus on Rwanda: Implications for Early Warning and Public Health Surveillance

**DOI:** 10.3390/pathogens15060574

**Published:** 2026-05-27

**Authors:** Sylvie Bambara, Marie Claire Isingizwe, Taofeek Tope Adegboyega, Leon Mutesa

**Affiliations:** 1 Department of Biomedical Sciences, School of Health Sciences, College of Medicine and Health Sciences, University of Rwanda (UR), Remera Campus, Kigali P.O. Box 4285, Rwanda; sylviebambara@gmail.com; 2 Inter Business Co., Ltd., Kigali P.O. Box 6683, Rwanda; claireisingizwe7@gmail.com; 3 Department of Microbiology & Parasitology, School of Medicine & Pharmacy, College of Medicine and Health Sciences, University of Rwanda (UR), Huye Campus, Kigali P.O. Box 4285, Rwanda; t.adegboyega@ur.ac.rw; 4 Center for Human Genetics and Genomics, College of Medicine and Health Sciences, University of Rwanda, Kigali P.O. Box 4285, Rwanda

**Keywords:** wastewater-based epidemiology, wastewater surveillance, environmental surveillance, pathogen detection, Africa, Rwanda, Kigali, SARS-CoV-2, AMR, virus detection, public health surveillance

## Abstract

In Africa, the disease burden of diarrheal and respiratory diseases is amplified by limited surveillance capacity, diagnostic limitations, and socioeconomic inequalities. In rapidly urbanizing settings such as Kigali (Rwanda), integrating wastewater-based epidemiology (WBE) into existing surveillance systems offers a promising strategy for generating real-time epidemiological intelligence, identifying community-level hotspots, and addressing gaps in traditional reporting systems. Gastrointestinal and respiratory infections remain major causes of morbidity and mortality globally, particularly in low- and middle-income countries (LMICs), where traditional clinical surveillance systems frequently underreport the true disease burden. This systematic review synthesizes current evidence on the detection of gastrointestinal and respiratory pathogens in wastewater and evaluates the utility of WBE for early warning and public health action. A narrative review approach was used to identify peer-reviewed literature, global health reports, and surveillance studies focusing on the wastewater detection of gastrointestinal and respiratory pathogens. Databases including PubMed, Scopus, and Google Scholar were searched for studies published between 2000 and 2026. The search yielded 1247 records, of which 312 duplicates were removed. After title/abstract screening, 228 full-text articles were retrieved and assessed for eligibility. After a detailed evaluation, 108 studies were excluded for the following reasons: absence of pathogen-specific wastewater data (*n* = 46), a focus on environmental monitoring without public health relevance (*n* = 25), insufficient methodological description (*n* = 21), or other eligibility limitations such as a lack of primary data (*n* = 16). WBE provides a non-invasive, cost-effective approach for monitoring symptomatic and asymptomatic infections. Challenges involve variability in sampling, environmental factors affecting viral decay, and differences in laboratory workflows. WBE is a powerful complement to traditional infectious disease surveillance, offering early warning capabilities, population-level coverage, and real-time insights into pathogen circulation. Integrating WBE into surveillance programs, especially in LMICs such as Rwanda, can significantly strengthen epidemic preparedness, guide resource allocation, and improve outbreak response. Sustained investment in laboratory capacity, standardized protocols, and multisector collaboration is essential to fully leverage WBE for public health protection.

## 1. Introduction

Wastewater-based epidemiology (WBE) has emerged as a powerful, non-invasive tool for monitoring infectious diseases at the community level, offering timely and cost-effective insights into pathogen circulation [1,2,3]. Globally, gastrointestinal and respiratory infections remain among the most significant public health threats, severely affecting populations in low- [4,5,6,7] and middle-income countries (LMICs) due to rapid population growth, urbanization, climate change, stressed sanitation systems, and rising antimicrobial resistance. These factors contribute to the spread and re-emergence of enteric and respiratory pathogens, generating an urgent need for advanced surveillance systems capable of detecting both symptomatic and asymptomatic infections. Traditional clinical surveillance, reliant on healthcare-seeking behavior, laboratory diagnostics, and reporting systems, captures only a portion of true cases, particularly in resource-limited settings where diagnostic coverage is uneven and health-seeking patterns vary widely [8,9,10]. Throughout the COVID-19 pandemic, delays in detecting community transmission and reliance on clinical testing critically damaged response efforts and intensified transmission in many regions [11,12,13]. These limitations have led to increasing global interest in environmental surveillance approaches, particularly WBE, as complementary tools for assessing pathogen transmission dynamics and strengthening epidemic preparedness in countries such as Rwanda. WBE involves analyzing sewage for genetic material or biomarkers shed by infected individuals, allowing the detection of both symptomatic and asymptomatic infections at a population level [3,13,14]. This approach has demonstrated strong performance in tracking a wide range of pathogens, including SARS-CoV-2, norovirus, rotavirus, hepatitis A virus, *Campylobacter* spp., *Salmonella* spp., and protozoan parasites such as *Giardia* and *Cryptosporidium* [15,16,17,18,19,20], to name a few. Numerous studies have demonstrated strong correlations between pathogen concentrations in wastewater and clinical case trends, with wastewater signals often preceding clinical waves by several days to weeks, highlighting the value of WBE as an early warning system [17,20,21,22,23]. Gastrointestinal pathogens, including *Escherichia coli*, *Salmonella*, *Vibrio cholerae*, norovirus, rotavirus, and hepatitis A virus, remain major contributors to global morbidity and mortality, mostly among children under five in LMICs [6,18,19,24]. In addition, respiratory pathogens, especially SARS-CoV-2 and influenza viruses, have been identified in wastewater, supporting the use of WBE for respiratory disease surveillance [12,22,25]. Wastewater monitoring consequently offers a critical opportunity to detect outbreaks, monitor endemic disease patterns, and evaluate the effectiveness of public health interventions, such as vaccination programs and improvements in water and sanitation infrastructure [17,24,26]. In Africa, the disease burden of diarrheal and respiratory diseases is amplified by limited surveillance capacity, diagnostic limitations, and socioeconomic inequalities [7,27,28]. In rapidly urbanizing settings such as Kigali (Rwanda), integrating WBE into existing surveillance systems is a promising strategy for generating real-time epidemiological intelligence, identifying community-level hotspots, and addressing gaps in traditional reporting systems [29,30]. Evidence from the COVID-19 pandemic revealed that WBE can detect emerging variants, identify resurgences even when clinical testing fails, and support data-driven decision-making in resource-limited environments [12,30,31,32]. Given these advantages, understanding how WBE can be leveraged for multi-pathogen detection in Kigali and similar urban centers is vital for strengthening public health preparedness and response. This systematic review synthesizes up-to-date evidence on the detection of gastrointestinal and respiratory pathogens in wastewater, evaluates relationships between wastewater pathogen concentrations and clinical case patterns, and emphasizes the role of WBE as an early warning tool. Furthermore, we discuss methodological considerations, challenges, and opportunities for integrating WBE into national disease surveillance frameworks in Rwanda and within Africa.

## 2. Materials and Methods

### 2.1. Protocol Registration

The protocol for this systematic review was registered in the PROSPERO International Prospective Register of Systematic Reviews (PROSPERO 2026: CRD420261358968). Although registration was not mandatory, the study employed a narrative synthesis rather than a quantitative meta-analysis. The review methods were established in advance and applied consistently throughout the study.

### 2.2. Study Selection Process (PRISMA Flow Diagram)

The study selection process followed the PRISMA 2020 guidelines [33], conducted through four stages: identification, screening, eligibility, and inclusion. The PRISMA 2020 checklist is provided as Supplementary Materials (Table S1) and cited here to ensure transparency and completeness of reporting.

**Figure 1:**
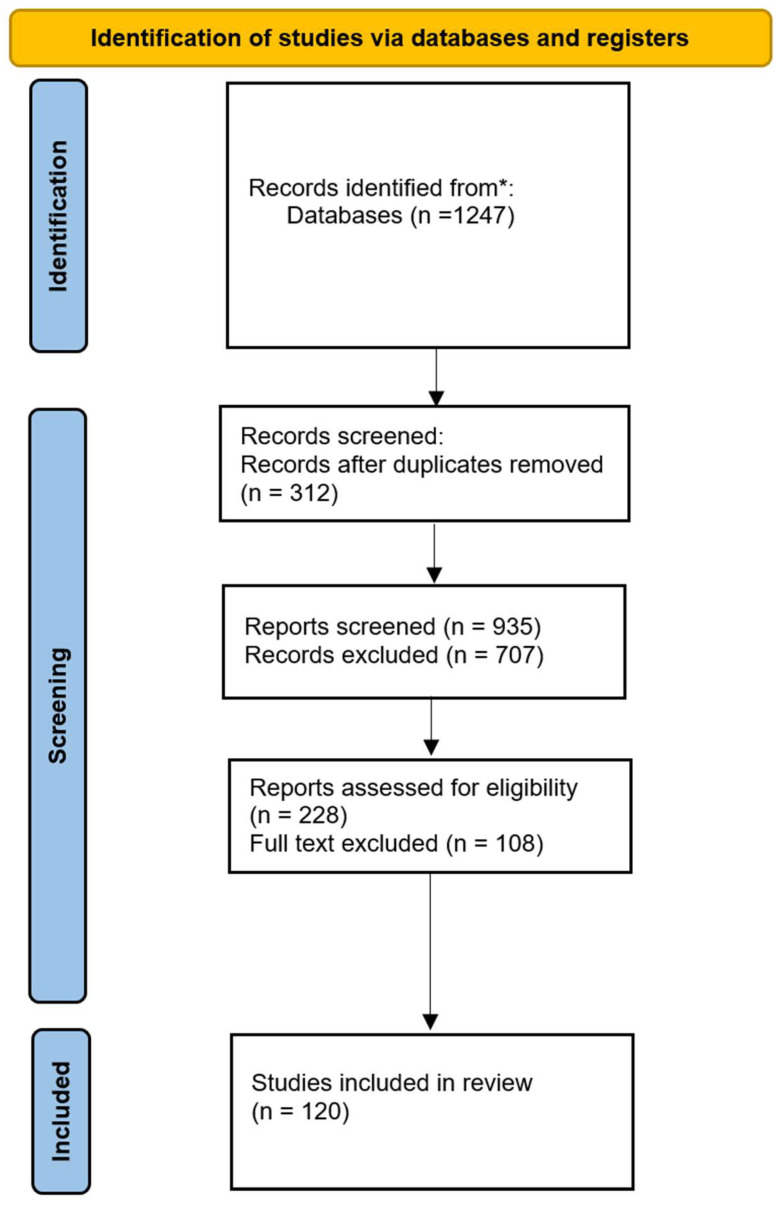
PRISMA flow chart: details of screening procedure.

All records identified through database searches were imported into reference management software, where duplicate records were identified and removed. The remaining articles were then subjected to title and abstract screening to assess their relevance to the objectives of the review. Studies were excluded at this stage if they were clearly unrelated to wastewater-based epidemiology, pathogen detection in wastewater, or public health surveillance. Articles considered potentially relevant were retrieved for full-text evaluation. During the eligibility stage, the full texts were carefully assessed against the predefined inclusion criteria, including studies reporting the detection, monitoring, or surveillance of gastrointestinal or respiratory pathogens in wastewater using validated analytical or molecular methods. Studies were excluded if they did not provide pathogen-specific wastewater data, focused exclusively on environmental monitoring without public health relevance, lacked sufficient methodological description, or did not present primary research data. Following the full-text assessment, 120 studies met the eligibility criteria and were included in the final qualitative synthesis. The complete study selection process is presented in Figure 1 (PRISMA flow diagram).

### 2.3. Why the African Context Matters

In Africa, with over 50 countries and rapidly expanding cities, the urban context of cities such as Kigali is critically important for advancing wastewater-based epidemiology (WBE). Many African countries experience an uneven burden of infectious diseases and frequent outbreaks, while surveillance systems often face diagnostic delays due to reduced laboratory capacity and the underreporting of clinical cases [34,35]. Rapid population growth, densely populated settlements, and uneven health infrastructure further heighten the risk of silent transmission, underscoring the need for early detection [36]. Kigali, Rwanda’s capital and largest city, with an estimated two million inhabitants according to the 2022 Rwandan Census, illustrates a rapidly developing urban environment where expanding wastewater networks and high population mobility create both challenges and opportunities for implementing WBE as a cost-effective surveillance tool [34,37].

In addition, WBE can strengthen early warning systems, complement traditional surveillance, and support more equitable outbreak readiness across African settings to provide population-level, near-real-time pathogen insights [38,39]. This systematic review was conducted following PRISMA principles to safeguard transparency and reproducibility. Five electronic databases were searched: PubMed/MEDLINE, Scopus, Web of Science, and the African Journals Online (AJOL) platform. The search covered studies published between January 2000 and January 2026. A comprehensive Boolean search strategy combining Medical Subject Headings (MeSH) and free-text terms was applied. The core search included combinations of wastewater-based epidemiology, wastewater surveillance, environmental surveillance, pathogen detection, Africa, Rwanda, Kigali, SARS-CoV-2, enteric pathogens, AMR, virus detection, and public health surveillance [38]. This assessment aims to synthesize current data on wastewater-based epidemiology for early pathogen detection, with an emphasis on its applicability in African frameworks. The latest studies reveal that WBE can reliably detect viral, bacterial, and antimicrobial-resistant pathogens and provide actionable public health insights before clinical data become available [39,40]. Nonetheless, research coverage across Africa remains limited, despite its demonstrated feasibility in countries such as South Africa, Nigeria, Rwanda, and Senegal [40,41,42]. In consolidating global and Africa-specific results, this systematic review identifies methodological advances, data gaps, and integration opportunities for strengthening public health surveillance. Ultimately, it highlights the potential for WBE to serve as a scalable and sustainable early warning system capable of refining disease preparedness in resource-limited environments such as Kigali.

### 2.4. Inclusion Criteria

Studies were considered eligible for inclusion in this review if they reported either primary or secondary data on the detection of pathogens in wastewater. Eligible studies were required to present microbiological, genomic, molecular, or epidemiological outcomes significant to wastewater-based epidemiology (WBE). In addition, studies conducted in African settings were selected, as well as those specifying methodological frameworks applicable to low-resource or resource-limited settings. Peer-reviewed articles, preprints, technical reports, and governmental or institutional surveillance documents published in either English or French were incorporated to ensure comprehensive coverage of both scientific and policy-oriented evidence.

### 2.5. Exclusion Criteria

Studies were excluded from the review if they were dedicated exclusively to industrial discharge or environmental monitoring without direct relevance to public health. Publications lacking empirical laboratory records or epidemiological evidence were also excluded, as were studies that did not report specific pathogen detection outcomes in wastewater. Moreover, duplicate records, editorials, opinion pieces, interpretations, and conference abstracts without full methodological descriptions were rejected to preserve methodological rigor and data reliability.

### 2.6. Screening Process

All recovered records were imported into Zotero version 7.0 for de-duplication, after which two independent reviewers selected titles and abstracts. Full texts of potentially eligible studies were then assessed for inclusion. Any differences were resolved through consensus or adjudication by a third reviewer. Data extraction focused on key study types and outcomes, including the country, study setting, the specific pathogens targeted, and the sampling and analytical methods employed. Extracted outcomes involved pathogen detection rates, assessable measurements, and any reported relationships between wastewater signals and corresponding clinical or epidemiological data. Furthermore, information on the public health applications of wastewater-based epidemiology, such as early warning, surveillance integration, or outbreak response, was methodically collected.

### 2.7. Technical Workflow Overview

This section provides a high-level overview of the biological and analytical workflows commonly used in the wastewater-based epidemiology studies included in this review.

### 2.8. Sampling Workflow

Most studies conducted in Africa generally employed one of three primary wastewater-sampling strategies. Grab sampling was usually used at influent points of wastewater treatment plants or at sewer junctions due to its operational simplicity. Composite sampling, frequently implemented using automated samplers, was used to integrate flow-proportional or time-proportional samples over a 24 h period to better capture temporal variability in pathogen shedding. Passive sampling approaches, involving Moore swabs and other absorbent materials, have gained increasing attention, especially in low-resource settings such as Rwanda, where they offer a cost-effective and logistically feasible alternative while maintaining analytical sensitivity [43]. Sample preservation normally involved cooling at 4 °C during transport, with laboratory processing performed within 6–24 h to minimize nucleic acid degradation and ensure data quality.

### 2.9. Concentration and Extraction Workflow

Viral and bacterial targets in wastewater samples were concentrated prior to analysis, utilizing a range of conventional methods. Normally applied approaches involved polyethylene glycol (PEG) precipitation, ultrafiltration, electronegative membrane adsorption–elution techniques, and flocculation-based protocols. The concentration method varied according to target pathogen type, available laboratory infrastructure, sample matrix, and resource settings, with each methodology offering distinct advantages in terms of recovery efficiency, scalability, and operational feasibility. RNA and DNA extractions were performed using commercial kits (e.g., Qiagen: Hilden, Germany Promega; Promega Maxwell: Corporation, Madison, WI, USA; ThermoFisher: Scientific, Waltham, MA, USA) or phenol-chloroform-based protocols, depending on laboratory capacity. Internal process controls, such as murine hepatitis virus (MHV), pepper mild mottle virus (PMMoV), and MS2 bacteriophage, were often used to gauge recovery efficiency and for normalization purposes [43].

### 2.10. Biological and Molecular Detection Workflow

Following nucleic acid extraction, pathogen detection and quantification were primarily performed by reverse transcription quantitative PCR (RT-qPCR) or quantitative PCR (qPCR) for viral and bacterial targets. Droplet digital PCR (ddPCR) was also used in several studies to achieve higher analytical sensitivity and enhanced absolute quantification, particularly for low-abundance targets. In addition, amplicon-based or whole-genome sequencing approaches were used to support variant tracking, antimicrobial resistance profiling, and phylogenetic inference. To increase data quality and comparability across sampling sites and periods, normalization and quality control strategies were applied, including the use of fecal indicators such as pepper mild mottle virus (PMMoV) and crAssphage, as well as adjustments based on wastewater flow rates [40,44]. This workflow reflects the methodologies used both globally and in emerging African studies, including Rwanda’s SARS-CoV-2 wastewater surveillance pilot in Kigali, which demonstrated strong concordance between wastewater viral concentrations and reported clinical trends.

## 3. Results

### 3.1. Prisma Layout Flow Summary

The literature search identified 1247 records across the selected databases. After removing 312 duplicate records, 935 unique articles remained for title and abstract screening.

During the screening stage, 707 records were excluded because they did not meet the predefined inclusion criteria. These exclusions primarily included articles focusing exclusively on environmental monitoring without pathogen detection, as well as studies unrelated to public health surveillance of gastrointestinal or respiratory pathogens. Following this step, 228 full-text articles were retrieved and assessed for eligibility. After a detailed evaluation, 108 studies were excluded; ultimately, 120 studies were included in this review.

### 3.2. Detection of Gastrointestinal Pathogens in Wastewater

Multiple studies have revealed that wastewater is a reliable and informative matrix for the detection of gastrointestinal pathogens circulating within communities. Viral agents frequently include norovirus, a leading global cause of acute gastroenteritis; rotavirus, which is particularly prevalent among children under five years of age; and hepatitis A virus, especially during community-level outbreaks. In addition to viral pathogens, wastewater surveillance has consistently detected a range of enteric bacteria such as *Escherichia coli*, *Campylobacter* spp., *Salmonella* spp., and *Vibrio cholerae*. Protozoan pathogens, including *Cryptosporidium* and *Giardia*, have also been identified, further highlighting the capacity of wastewater-based epidemiology to capture diverse gastrointestinal pathogens of public health importance. Detection rates vary by pathogen type, environmental conditions, and sampling approach, but wastewater monitoring consistently tracks pathogen circulation even when clinical reporting is low.

Table 1 provides a consolidated overview of wastewater-based epidemiology (WBE) initiatives conducted across the African continent. These studies vary extensively in scope from SARS-CoV-2 surveillance and poliovirus environmental monitoring to antimicrobial resistance (AMR) detection and enteric virus tracking. The table highlights the variety of pathogens monitored, methodological platforms used, and the uneven distribution of WBE activities across countries. It also highlights the growing role of WBE as a complementary surveillance tool in resource-limited settings.

Table 2 summarizes peer-reviewed studies that applied wastewater-based epidemiology methods across African countries, including study focus, pathogens detected, analytical platforms, and reference sources. It synthesizes key gastrointestinal pathogens detected in wastewater and the platforms commonly used for their identification. Enteric viruses such as norovirus, rotavirus, and adenovirus remain the most reported organisms due to their high shedding rates and environmental stability. Bacterial and protozoan pathogens require more specialized methods, including culture, PCR, and immunofluorescence assays. This table provides a technical foundation for understanding how wastewater reflects community-level circulation of GI diseases. All studies included were published between January 2000 and January 2026.

### 3.3. Detection of Respiratory Pathogens in Wastewater

Respiratory virus detection in wastewater has increased rapidly since the COVID-19 pandemic, providing critical insights into community-level transmission dynamics. Several studies have shown that SARS-CoV-2 RNA is highly detectable in wastewater and demonstrates strong correlations with reported clinical case trends. Importantly, wastewater viral signals have normally been observed to precede clinical case surges by approximately 4 to 10 days, underscoring the early warning potential of wastewater-based epidemiology. In addition to SARS-CoV-2, influenza viruses (A and B) have also been effectively identified in untreated wastewater, further supporting the feasibility of wastewater surveillance for monitoring respiratory virus circulation beyond pandemic settings. These findings underscore the relevance of WBE beyond gastrointestinal pathogens, supporting integrated multi-pathogen surveillance systems.

### 3.4. Correlations Between Wastewater Signals and Clinical Case Data

Strong correlations between pathogen concentrations measured in wastewater and reported clinical cases have been demonstrated across diverse geographic regions. Wastewater signals of SARS-CoV-2 have been shown to closely track, and frequently forecast, community infection trends. Likewise, increases in norovirus RNA concentrations in wastewater coincide with gastroenteritis outbreaks, while rotavirus detection patterns align with seasonal trends in pediatric disease. Detection of hepatitis A virus in sewage has also been reported to precede clinical case spikes, enabling early outbreak identification. These correlations reinforce the value of wastewater-based epidemiology as a sensitive and timely population-level surveillance tool for infectious disease monitoring.

The studies included in Table 3 were selected for their methodological rigor, the clarity of their wastewater detection outcomes, and their relevance to respiratory pathogen surveillance. The table also differentiates studies that used qPCR for DNA-based targets from those that employed RT-qPCR for RNA viruses.

Table 3 summarizes respiratory pathogens that have been effectively identified in wastewater and stresses the rationale for their environmental detectability. SARS-CoV-2 remains the most documented respiratory virus in wastewater systems, but emerging evidence shows that influenza viruses, RSV, and adenoviruses can also be detected. This demonstrates the expanding utility of WBE beyond enteric diseases, offering potential for integrated respiratory pathogen surveillance, especially during outbreaks or seasonal peaks. In addition, a summary comparison of RT-qPCR and sequencing approaches is presented in Table 3.

### 3.5. Technical and Operational Considerations

The quality and reliability of wastewater-based epidemiology data are influenced by several interrelated methodological factors. These involve the choice of sampling strategy, such as grab versus composite sampling, and the use of raw sewage compared with sludge, which can affect pathogen recovery and temporal representativeness. RNA preservation practices and viral decay dynamics throughout collection, transport, and storage further influence detection sensitivity. In addition, variations in extraction and concentration workflows can considerably impact nucleic acid yield and analytical performance. The selection of molecular detection methods, including RT-qPCR, droplet digital PCR (ddPCR), and metagenomic sequencing, also determines sensitivity, quantification accuracy, and pathogen coverage. Finally, normalization approaches, such as the use of fecal indicators (e.g., PMMoV), wastewater flow data, or chemical tracers, are critical for improving comparability across sampling sites and over time.

Examples of studies demonstrating strong correlations between wastewater pathogen concentrations and reported clinical cases are summarized in Table 4. Across diverse settings, these studies consistently show that changes in wastewater signals often precede increases in clinically reported incidence by several days to weeks, underscoring the value of wastewater-based epidemiology (WBE) as an early warning system. This observed strength of correlation supports the integration of WBE into national disease surveillance frameworks, particularly in contexts where access to timely diagnostic testing is limited (Table 4).

A comparison of the major advantages and limitations of wastewater-based epidemiology in public health surveillance is shown. Table 5 outlines the major strengths and limitations of WBE as reported in the scientific literature. While wastewater surveillance offers early detection, population-wide coverage, and the capability to capture asymptomatic infections, it also faces methodological and operational challenges. These include viral decay, infrastructure variability, and limitations in quantification methods. Understanding these advantages and constraints is essential for designing feasible and impactful WBE programs in low-resource contexts.

Table 6 summarizes the principal barriers limiting wastewater-based epidemiology (WBE) implementation in low-resource African settings and outlines practical, context-appropriate strategies to address them. Key constraints include fragmented sewerage infrastructure, limited laboratory and workforce capacity, lack of standardized protocols, weak integration with public health surveillance systems, and challenges related to sustainable financing, governance, and data interpretation.

### 3.6. Regional WBE Evidence in Africa with Focus on Rwanda

To position the findings of this study within the broader African context, Table 7 (summary table of wastewater-based epidemiology studies in Africa) provides a consolidated overview of WBE initiatives conducted across the continent. These studies demonstrate how wastewater surveillance has been applied to detect a wide range of pathogens, including SARS-CoV-2, poliovirus, enteric viruses, antimicrobial-resistant bacteria, and viral hepatitis, using diverse analytical platforms such as RT-qPCR, cell culture, PEG precipitation, metagenomic sequencing, and passive environmental sampling. The regional data highlights numerous trends relevant when considering Rwanda’s progress. Countries with long-standing environmental surveillance systems, such as South Africa, Nigeria, and Senegal, have leveraged their existing laboratory networks to rapidly integrate SARS-CoV-2 wastewater monitoring, demonstrating strong correlations with clinical case trends and generating real-time public health insights. In contrast, countries such as Ghana, Kenya, Uganda, and Ethiopia have applied WBE to target specific public health priorities, including enteric viruses, antimicrobial resistance, and bacterial pollution in wastewater systems.

Alongside this background, Rwanda’s recent expansion of WBE activities aligns with wider regional improvements while also demonstrating notable innovation. Rwanda’s community wastewater surveillance, combined with the development of WBE frameworks for early outbreak detection and the integration of airport-based genomic surveillance using aircraft wastewater, places the country among Africa’s emerging leaders in leveraging wastewater as a strategic public health tool. These initiatives are particularly significant in a situation where conventional clinical surveillance may face constraints related to access, testing coverage, and resource limitations.

The evidence summarized in Table 7 shows that WBE is gradually becoming recognized across Africa as a complementary and cost-effective surveillance approach. Comparing Rwanda’s experience with other countries highlights both shared challenges, such as laboratory capacity, infrastructure variability, and resource constraints, and substantial opportunities for strengthening national and regional disease early warning systems through consistent WBE practices, integrated genomic surveillance, and cross-country knowledge exchange.

**Figure 2:**
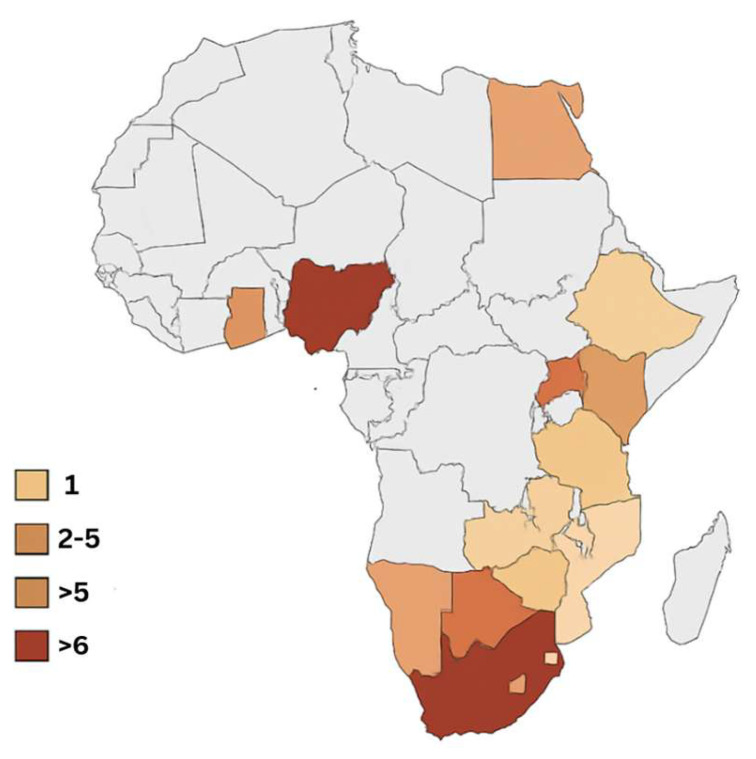
Wastewater-based epidemiology studies in Africa. Countries represented include South Africa, Nigeria, Rwanda, Senegal, Ghana, Kenya, Uganda, Ethiopia, Egypt, Tunisia, Morocco, and Zimbabwe. Gray areas represent African countries where no wastewater-based epidemiology (WBE) studies meeting the inclusion criteria were identified or reported.

Figure 2, in combination with Table 7, shows the geographic distribution, thematic focus, and methodological diversity of wastewater-based epidemiology (WBE) studies conducted across Africa. The figure highlights that WBE activities have been concentrated in a limited number of countries, with South Africa, Nigeria, and Rwanda emerging as regional leaders in the implementation of wastewater surveillance. South Africa demonstrates the most extensive WBE portfolio, including SARS-CoV-2 surveillance, long-standing poliovirus environmental surveillance, and antimicrobial resistance (AMR) monitoring using advanced molecular platforms, with RT-qPCR, metagenomic sequencing, and PEG-based concentration methods [76,77]. Several countries, including Nigeria, Senegal, and Ghana, have leveraged WBE mainly for enteric virus surveillance, particularly poliovirus and other gastrointestinal pathogens, reflecting alignment with global polio eradication and diarrheal disease control initiatives [78,79]. In contrast, North African countries such as Egypt, Tunisia, and Morocco have focused predominantly on SARS-CoV-2 and hepatitis virus detection in wastewater, largely adopting RT-qPCR-based approaches during the COVID-19 pandemic [80,81]. East African countries, including Kenya, Uganda, Ethiopia, and Rwanda, demonstrate an increasing integration of WBE into antimicrobial resistance surveillance and pandemic preparedness. Kenya and Uganda have applied culture-based approaches combined with PCR to reveal resistant *Escherichia coli*, *Enterococcus*, and other clinically significant bacteria [82,83], while Ethiopia has focused on quantifying resistance genes using qPCR [73]. Rwanda presents a particularly notable case, with several complementary WBE initiatives ranging from municipal SARS-CoV-2 surveillance to the development of national wastewater surveillance frameworks and innovative airport-based wastewater and genomic surveillance targeting imported variants at Kigali International Airport [27,29,47].

## 4. Discussion

### 4.1. Overview of Wastewater-Based Epidemiology (WBE) for GI and Respiratory Pathogens

Wastewater-based epidemiology (WBE) has emerged as a powerful population-level surveillance tool capable of detecting a broad spectrum of gastrointestinal (GI) and respiratory pathogens shed in human excreta. Pathogens excreted in feces, urine, saliva, sputum, and mucus enter sewage networks, allowing the aggregation of biological signals from large communities regardless of healthcare-seeking behavior or testing access [84,85]. WBE complements traditional surveillance by providing near-real-time data on pathogen trends, including viruses (e.g., norovirus, rotavirus, adenovirus, and SARS-CoV-2), bacteria (e.g., *Salmonella*, *Shigella*, and *Vibrio*), and antimicrobial resistance (AMR) markers [86,87,88]. Its application expanded substantially during the COVID-19 pandemic and is currently recognized as a scalable early warning approach, particularly valuable for resource-limited settings where routine clinical surveillance remains fragmented [37,89].

### 4.2. Detection of Gastrointestinal Pathogens in Wastewater

Various studies have demonstrated successful detection of GI pathogens in wastewater using RT-qPCR, ddPCR, culture-based assays, and sequencing. Enteric viruses such as norovirus, rotavirus, hepatitis A virus (HAV), and enteroviruses are among the most frequently monitored and consistently detected at high concentrations due to robust fecal shedding dynamics [90,91,92]. Bacterial pathogens, including *Salmonella enterica*, *Vibrio cholerae*, *Shigella* spp., *Campylobacter* spp., and diarrheagenic *Escherichia coli*, have been identified across various wastewater systems globally, including in African settings such as South Africa, Nigeria, Senegal, and Rwanda [93,94]. Wastewater has also proven valuable for identifying AMR genes (e.g., *bla*CTX-M, carbapenemases, and macrolide-resistance markers), offering insights into community-level resistance pressures [45]. These findings highlight WBE’s strong potential to track GI diseases that represent a major burden across Africa.

### 4.3. Detection of Respiratory Pathogens in Wastewater

Although respiratory pathogens are not naturally associated with fecal–oral transmission, several respiratory viruses, including SARS-CoV-2, influenza A/B, respiratory syncytial virus (RSV), and human metapneumovirus, have been consistently detected in wastewater due to mucosal shedding and enteric replication pathways [95,96]. SARS-CoV-2 surveillance via WBE has been broadly validated, including in low-resource settings such as Kigali, where viral RNA trends strongly mirrored clinical case patterns during multiple pandemic waves [97,98]. Emerging studies also demonstrate wastewater detection of influenza viruses and RSV, enabling population-level monitoring even when clinical testing capacity decreases or asymptomatic infections dominate transmission [99]. These advances expand the scope of WBE from traditional GI pathogens toward broader respiratory disease surveillance.

### 4.4. Correlation Between Wastewater Signals and Clinical Data

A reliable finding across the global literature is the strong correlation between wastewater pathogen concentrations and reported clinical case data. For SARS-CoV-2, multiple studies demonstrated that increases in wastewater RNA concentrations preceded rises in clinical cases by 4 to 14 days, making WBE a dependable early warning indicator [22,100,101]. Similar correlations have been observed for norovirus, rotavirus, poliovirus, hepatitis A, and *Salmonella* outbreaks, where wastewater detection also predicted or reflected community transmission dynamics [102,103]. In Rwanda, wastewater SARS-CoV-2 concentrations measured at major treatment facilities in Kigali showed high concordance with PCR-confirmed case data throughout the pandemic [104]. This alignment emphasizes WBE’s utility in settings with limited diagnostic coverage, asymptomatic infections, or delays in clinical reporting.

### 4.5. Strengths and Limitations of WBE in Public Health Surveillance

Wastewater-based epidemiology (WBE) offers several strengths that make it a valuable tool for communicable disease surveillance. It is a cost-effective surveillance approach, particularly in low-resource settings where widespread individual testing is not feasible [1,105]. In addition, WBE allows the simultaneous monitoring of multiple pathogens, including antimicrobial resistance markers, and offers a non-invasive, community-wide surveillance mechanism that supports equity in public health responses [106]. It also provides population-level coverage that is independent of healthcare-seeking behavior, allowing more comprehensive monitoring of community infection dynamics [107]. WBE can detect pathogen circulation early, often before symptomatic cases peak, thereby supporting suitable public health interventions [87,99]. Despite these benefits, WBE also presents several limitations. Variability in sewage infrastructure can affect the representativeness of samples, particularly in informal or underserved settlements [108,109]. Environmental factors such as temperature, pH, flow rate, and hydraulic retention time influence the decay of RNA and DNA in wastewater, potentially decreasing detection sensitivity [110]. Further challenges include difficulties in accurate quantification, normalization, and establishing direct links between wastewater signals and precise case numbers [111]. Furthermore, limited laboratory capacity for molecular analysis and sequencing in many African regions constrains large-scale implementation [112]. However, when integrated with environmental, clinical, and genomic data, WBE remains a powerful and complementary approach to traditional surveillance systems, particularly for strengthening early warning and public health awareness.

### 4.6. Implications for Early Warning Systems

WBE’s capacity to identify pathogens before clinical increases occur makes it a cornerstone for early warning systems. Through the detection of pre-symptomatic and asymptomatic shedding, WBE can guide rapid interventions such as targeted screening, vaccination campaigns, public risk communication, and resource allocation [22,87]. During the COVID-19 pandemic, WBE allowed early detection of emerging variants and informed public health decision-making even when clinical testing capacity was overwhelmed [22,113]. Similar frameworks are now being applied to other pathogens, such as cholera, norovirus, and AMR genes, to support outbreak prediction and response strategies in Africa and other resource-limited regions [114,115]. Its integration into national surveillance systems can significantly increase resilience against future outbreaks.

### 4.7. Opportunities for Implementation in Low-Resource African Settings

African settings offer strong potential for scaling wastewater-based epidemiology (WBE) due to the high burden of infectious diseases, limitations of clinical surveillance, and expanding urban wastewater infrastructure in cities such as Kigali, Nairobi, Johannesburg, Dakar, and Lagos [37,78]. Despite the demonstrated feasibility and public health value in several African countries, sustainable scale-up remains constrained by structural, technical, and governance-related gaps. As summarized in Table 6, a major challenge is the heterogeneity and incompleteness of sewerage systems, particularly in informal and peri-urban settlements where centralized wastewater collection is limited [116]. In these contexts, passive sampling approaches (e.g., Moore swabs) provide low-cost, operationally feasible solutions and have demonstrated effectiveness for viral and bacterial detection in low-resource settings such as Rwanda and Senegal [117,118].

Additional barriers include limited laboratory capacity and workforce shortages, a lack of standardized protocols, weak integration of WBE outputs into public health surveillance systems, and challenges related to sustainable financing, governance, and data interpretation [119,120]. As outlined in Table 6, pragmatic mitigation strategies include phased laboratory capacity strengthening through regional reference laboratories and targeted training [121], adoption of harmonized standard operating procedures aligned with the WHO and Africa CDC frameworks [122], and the establishment of formal data-sharing mechanisms to support early warning and outbreak response. Sustainable expansion will also require integrating WBE into routine surveillance budgets and leveraging existing disease control platforms.

## 5. Conclusions

Wastewater-based epidemiology provides a powerful, non-invasive approach for monitoring the community-level circulation of gastrointestinal and respiratory pathogens. Evidence demonstrates that wastewater signals often precede clinical cases, suggesting valuable early warning for outbreaks. WBE is especially valuable in resource-limited settings where diagnostic access is constrained. Integrating WBE into national surveillance frameworks can enhance infectious disease monitoring, inform targeted interventions, and strengthen overall public health resilience. Continued investment in laboratory infrastructure, capacity building for early career researchers, standardized methodologies, and cross-sector collaboration will be essential to scaling WBE programs across Africa and globally.

## Figures and Tables

**Table 1:** Detection of gastrointestinal pathogens in wastewater.

Targeted Pathogen	Preferred Detection Method(s)	Reference
Enteric viruses	RT-qPCR, ddPCR	[18,19]
Norovirus, rotavirus, enteroviruses, HAV	Culture, qPCR, sequencing	[43,45]
Bacterial pathogens	Immunofluorescence, qPCR	[45]
AMR pathogens in sewage, *Salmonella* spp., *Shigella* spp., *Vibrio cholera*		
Protozoa		
*Giardia*, *Cryptosporidium*		
AMR markers *blaCTX-M*, *blaNDM*, macrolide-resistance genes	qPCR, metagenomic sequencing	[46]

**Table 2:** Summary of major pathogens detectable in wastewater.

Country	Study Focus	Pathogens Detected	Analytical Platform	Reference
Rwanda	SARS-CoV-2 wastewater surveillance and feasibility	SARS-CoV-2 (RNA)	RT-qPCR, passive sampling	[47]
South Africa	National WBE network for COVID-19	SARS-CoV-2, PMMoV	RT-qPCR, normalization	[48]
Nigeria	Enteric virus detection	Enteroviruses, adenovirus, norovirus	RT-PCR, cell culture	[49]
Senegal	Fecal sludge/viral persistence	Enteric and respiratory viruses	RT-qPCR	[42]
Egypt	Detection of HAV and HEV viruses	HAV, HEV	RT-PCR	[50]
South Africa	Bacterial pathogen tracking	*Salmonella*, *Shigella*, *Vibrio*	Culture + PCR	[43]

**Table 3:** Detection of respiratory pathogens in wastewater.

Pathogen	Rationale for Wastewater Detection	Analytical Platform	Reference
SARS-CoV-2	Fecal shedding; mucosal shedding	RT-qPCR, ddPCR, sequencing	[34]
Influenza A/B	Fecal shedding in infected individuals	RT-qPCR	[48]
RSV	Presence in stool and respiratory excretions	RT-qPCR	[48]
Human adenovirus	Dual GI and respiratory involvement	qPCR	[17]

**Table 4:** Correlation between wastewater signals and clinical cases.

Pathogen	Correlation Outcome	Setting	Reference
SARS-CoV-2	Wastewater signal increases preceding clinical cases by 4 to 14 days	Rwanda, Europe, USA	[37,47]
Norovirus	Wastewater peaks reflect seasonal occurrences	Sweden	[18]
Polio	Strong correlation between wastewater and AFP surveillance	Global (WHO)	[51]
Hepatitis A	Strong correlation between wastewater and AFP surveillance	Japan, Italy	[19]

**Table 5:** Strengths and limitations of WBE (this table complements the narrative discussion of strengths and limitations).

Category	Key Points	Reference
Strengths	Early detection, population-wide coverage, cost-effective, and includes asymptomatic infections	[37]
Limitations	Infrastructure gaps, variable viral decay, quantification challenges, and limited lab capacity	[37]

**Table 6:** Key gaps limiting WBE implementation in low-resource African settings, practical strategies, and supporting evidence.

Missing/Limiting Factor	Description in the African Context	Practical Strategies to Overcome the Gap	Key References
Fragmented or incomplete sewer networks	Many urban and peri-urban areas rely on on-site sanitation, informal drainage systems, or fecal sludge management, limiting the representativeness of centralized wastewater sampling.	Use passive sampling (e.g., Moore swabs) at strategic sewer junctions, pumping stations, open drains, and fecal sludge discharge points.	[42,43]
Limited laboratory molecular capacity	Shortage of RT-qPCR platforms, reagents, sequencing capacity, and skilled personnel for routine wastewater analysis.	Establish regional reference laboratories, shared sequencing hubs, phased laboratory strengthening, and south–south technical collaboration.	[45,51]
Lack of standardized protocols	Heterogeneity in sampling, concentration, normalization, and reporting reduces data comparability and policy confidence.	Adopt harmonized SOPs aligned with the WHO poliovirus environmental surveillance and the Africa CDC frameworks.	[40,51,52]
Weak integration with public health surveillance systems	Wastewater data is often generated through research projects without formal linkage to epidemiological decision-making.	Create formal data-sharing pathways with ministries of health, disease surveillance units, and public health emergency operation centers.	[32,34]
Insufficient sustainable financing	Reliance on short-term donors or research funding limits continuity and scale-up.	Integrate WBE into routine national surveillance budgets and leverage existing platforms (polio, AMR, and COVID-19 legacy systems).	[37,49,52]
Limited governance and regulatory frameworks	Absence of clear policies on data ownership, reporting responsibility, and ethical use of wastewater surveillance data.	Develop national WBE guidelines, governance frameworks, and ethical oversight under the Ministry of Health leadership.	[26,52]
Human resource constraints	Limited number of trained professionals in environmental microbiology, molecular diagnostics, and data interpretation.	Implement targeted training programs, integrate WBE into academic curricula, and strengthen workforce retention strategies.	[45]
Challenges in data interpretation and actionability	Difficulty translating pathogen concentrations into actionable public health thresholds.	Use trend-based indicators, triangulate wastewater signals with clinical data, and develop early warning dashboards.	[31]

**Table 7:** Wastewater-based epidemiology (WBE) studies in Africa.

Country	Research Topic	Disease/Pathogen Identified	Papers	Platform Used	Scientific References
South Africa	SARS-CoV-2 detection in municipal wastewater	COVID-19	6+	RT-qPCR, sequencing (illumine), PEG concentration	[53]
	Poliovirus environmental surveillance	Polio (WPV, cVDPV)	3	Cell culture, RT-PCR	[54,55]
	AMR genes in wastewater	Antimicrobial resistance	2	Metagenomics, qPCR	[49]
Nigeria	Poliovirus environnemental surveillance (national surveillance program)	Polio	5+	PCR, cell culture	[49,52,56]
	SARS-CoV-2 in wastewater	COVID-19	1	RT-qPCR	[46]
Egypt	Hepatitis A&E monitoring in wastewater	HAV, HEV	2	RT-PCR	[50,57]
	SARS-CoV-2 detection	COVID-19	1	RT-qPCR	[58]
Tunisia	SARS-CoV-2 detection in wastewater	COVID-19	2	RT-qPCR;	[59]
Morocco	Surveillance of SARS-CoV-2 in urban wastewater	COVID-19	1	RT-qPCR	[60]
Kenya	Antibiotic-resistant bacteria in wastewater	AMR bacteria (*E. coli*, *Klebsiella*, *Salmonella*)	2	Culture, AST, PCR	[61,62,63]
	SARS-CoV-2 wastewater surveillance	COVID-19	1	RT-qPCR	[63]
Uganda	AMR monitoring in wastewater	Resistance *E. coli* and *Enterococcus*	1	Culture-based PCR	[64]
Ghana	Environmental detection of enteric viruses	Norovirus, rotavirus, adenovirus	2	RT-PCR	[65,66]
Senegal	Polio environmental surveillance	Polioviruses	2	Cell culture, PCR	[42,67]
Zimbabwe	SARS-CoV-2 detection in municipal wastewater	COVID-19	3	RT-qPCR, viral concentration (PEG), and sequencing support via regional labs	[55,68,69]
Rwanda	Development of a wastewater surveillance framework for early outbreak detection	Multi-pathogen (SARS-CoV-2) enteric viruses	2	RT-qPCR, environmental sampling protocol	[37,53,70]
	Airport-based WBE and genomic surveillance using aircraft wastewater and pooled nasal swabs from international travelers at Kigali International Airport	SARS-CoV-2 and variants and other imported lineages	≥2	Aircraft wastewater sampling + pooled nasal swabs; RT-qPCR and whole-genome sequencing to detect imported variants; integration with national genomic surveillance	[27,29,37,38,47,70,71,72]
Ethiopia	AMR genes	ARGs (*bla*CTX-M, *mecA*, etc.)	1	qPCR	[73,74,75]

## Data Availability

No new data were created or analyzed in this study. Data sharing is not applicable to this article.

## Supplementary Materials

The following supporting information can be downloaded at: https://www.mdpi.com/article/10.3390/pathogens15060574/s1. Supplementary Table S1: PRISMA 2020 Checklist for reporting systematic reviews.

## Abbreviations

The following abbreviations are used in this manuscript:

Abbreviation	Definition
AMR	Antimicrobial Resistance
AMS	Antimicrobial Stewardship
API20E	Analytical Profile Index 20 Enterobacterales
ARGs	Antimicrobial Resistance Genes
AST	Antimicrobial Susceptibility Testing
CLSI	Clinical and Laboratory Standards Institute
CSF	Cerebrospinal Fluid
CCN	Clinical Case Notification
COVID-19	Coronavirus Disease 2019
ddPCR	Droplet Digital Polymerase Chain Reaction
DNA	Deoxyribonucleic Acid
ESBL	Extended-Spectrum Beta-Lactamase
GI	Gastrointestinal
GLASS	Global Antimicrobial Resistance Surveillance System
HAI	Hospital-Acquired Infection
HAV	Hepatitis A Virus
HEV	Hepatitis E Virus
ICU	Intensive Care Unit
IPC	Infection Prevention and Control
IRB	Institutional Review Board
LMICs	Low- and Middle-Income Countries
MDRO	Multidrug-Resistant Organism
MeSH	Medical Subject Headings
MIC	Minimum Inhibitory Concentration
MS2	MS2 Bacteriophage
PMMoV	Pepper Mild Mottle Virus
PRISMA	Preferred Reporting Items for Systematic Reviews and Meta-Analyses
qPCR	Quantitative Polymerase Chain Reaction
RNA	Ribonucleic Acid
RSV	Respiratory Syncytial Virus
RT-PCR	Reverse Transcription Polymerase Chain Reaction
RT-qPCR	Reverse Transcription Quantitative PCR
SARS-CoV-2	Severe Acute Respiratory Syndrome Coronavirus 2
SSIs	Surgical Site Infections
UN DESA	United Nations Department of Economic and Social Affairs
WHO	World Health Organization
WBE	Wastewater-Based Epidemiology
WPV	Wild Poliovirus
