# Supplementary material for: A Systematic Review of Gastrointestinal and Respiratory Pathogen Detection in Wastewater in Africa, with Focus on Rwanda: Implications for Early Warning and Public Health Surveillance
Source: Pathogens. 2026 May 27;15(6):574. doi: 10.3390/pathogens15060574 (PMC13304791; doi:10.3390/pathogens15060574)
Supplement: Supplementary file 1 [file pathogens-15-00574-s001.zip › pathogens-4243881-supplementary.pdf]

PRISMA 2020 Checklist

Section and Topic	Item #	Checklist item	Location where item is reported
**TITLE**: A Review of Gastrointestinal and Respiratory Pathogen Detection in Wastewater in Africa, with Focus in Rwanda: Implications for Early Warning and Public Health Surveillance			
Title	1	Identify the report as a systematic review.	Title page
**ABSTRACT**			
Abstract	2	See the PRISMA 2020 for Abstracts checklist.	Abstract
**INTRODUCTION**			
Rationale	3	Describe the rationale for the review in the context of existing knowledge.	Introduction (Section 1)
Objectives	4	Provide an explicit statement of the objective(s) or question(s) the review addresses.	Introduction (Section 1, final paragraph)
**METHODS**			
Eligibility criteria	5	Specify the inclusion and exclusion criteria for the review and how studies were grouped for the syntheses.	Methods (Sections 2.4 and 2.5)
Information sources	6	Specify all databases, registers, websites, organisations, reference lists and other sources searched or consulted to identify studies. Specify the date when each source was last searched or consulted.	Methods (Sections 2.3 and 2.2); databases: PubMed/MEDLINE, Scopus, Web of Science, AJOL, Google Scholar; search period: January 2000 to January 2026
Search strategy	7	Present the full search strategies for all databases, registers and websites, including any filters and limits used.	*The full search strategy was developed using a combination of Medical Subject Headings (MeSH) and free-text terms related to wastewater-based epidemiology, pathogen detection, and public health surveillance. The core search string included combinations of terms such as: "wastewater-based epidemiology", "wastewater surveillance", "environmental surveillance",*

PRISMA 2020 Checklist

Section and Topic	Item #	Checklist item	Location where item is reported
			"pathogen detection", "Africa", "Rwanda", "Kigali", "SARS-CoV-2", "enteric pathogens", "antimicrobial resistance", and "public health surveillance". Boolean operators (AND/OR) were used to combine terms. Due to database-specific differences, exact search strings were adapted accordingly. Although full search strings were not included in the manuscript, the search approach was consistently applied across all databases.
Selection process	8	Specify the methods used to decide whether a study met the inclusion criteria of the review, including how many reviewers screened each record and each report retrieved, whether they worked independently, and if applicable, details of automation tools used in the process.	Methods (Section 2.6): two independent reviewers; disagreements resolved by consensus or third reviewer
Data collection process	9	Specify the methods used to collect data from reports, including how many reviewers collected data from each report, whether they worked independently, any processes for obtaining or confirming data from study investigators, and if applicable, details of automation tools used in the process.	Methods (Section 2.6): two reviewers extracted data; key variables listed
Data items	10a	List and define all outcomes for which data were sought. Specify whether all results that were compatible with each outcome domain in each study were sought (e.g. for all measures, time points, analyses), and if not, the methods used to decide which results to collect.	Methods (Section 2.6): outcomes include pathogen detection rates, measurable measurements, and relationships between wastewater signals and clinical/epidemiological data

PRISMA 2020 Checklist

Section and Topic	Item #	Checklist item	Location where item is reported
	10b	List and define all other variables for which data were sought (e.g. participant and intervention characteristics, funding sources). Describe any assumptions made about any missing or unclear information.	Methods (Section 2.6): variables include country, study setting, pathogens targeted, sampling and analytical methods; public health applications
Study risk of bias assessment	11	Specify the methods used to assess risk of bias in the included studies, including details of the tool(s) used, how many reviewers assessed each study and whether they worked independently, and if applicable, details of automation tools used in the process.	*A formal risk of bias assessment tool (e.g., ROBIS or AMSTAR) was not applied due to the narrative nature of this review and the heterogeneity of included study designs. Instead, methodological quality was considered during study selection by excluding studies lacking sufficient methodological detail or primary data. The review acknowledges variability in study quality as a limitation*
Effect measures	12	Specify for each outcome the effect measure(s) (e.g. risk ratio, mean difference) used in the synthesis or presentation of results.	*Effect measures such as risk ratios or mean differences were not applicable, as this review followed a qualitative narrative synthesis approach without quantitative meta-analysis. Outcomes were described descriptively based on reported findings across studies.*
Synthesis methods	13a	Describe the processes used to decide which studies were eligible for each synthesis (e.g. tabulating the study intervention characteristics and comparing against the planned groups for each synthesis (item #5)).	Methods (Section 2.2 and 2.6): eligibility assessed through four PRISMA stages;

Section and Topic	Item #	Checklist item	Location where item is reported
			studies grouped by pathogen type (GI vs. respiratory)
	13b	Describe any methods required to prepare the data for presentation or synthesis, such as handling of missing summary statistics, or data conversions.	*No formal data transformation or statistical handling of missing data was performed. Extracted data were synthesized narratively. Variations in reporting across studies were addressed through descriptive comparison and thematic grouping.*
	13c	Describe any methods used to tabulate or visually display results of individual studies and syntheses.	Results (Sections 3.1-3.6): data presented in summary tables (Tables 1-7) and a PRISMA flow diagram (Figure 1)
	13d	Describe any methods used to synthesize results and provide a rationale for the choice(s). If meta-analysis was performed, describe the model(s), method(s) to identify the presence and extent of statistical heterogeneity, and software package(s) used.	Methods (Section 2.3): narrative synthesis approach without quantitative meta-analysis; rationale stated as heterogeneity of study designs. No statistical model described.
	13e	Describe any methods used to explore possible causes of heterogeneity among study results (e.g. subgroup analysis, meta-regression).	*No formal subgroup analysis or meta-regression was conducted to explore heterogeneity due to the qualitative nature of the review. However, heterogeneity across studies was considered descriptively in terms of differences in study*

Section and Topic	Item #	Checklist item	Location where item is reported
			settings, pathogens, and methodological approaches.
	13f	Describe any sensitivity analyses conducted to assess robustness of the synthesized results.	*Sensitivity analyses were not conducted, as the review did not include quantitative synthesis or meta-analysis. This is consistent with the narrative design of the study.*
Reporting bias assessment	14	Describe any methods used to assess risk of bias due to missing results in a synthesis (arising from reporting biases).	*Formal assessment of reporting bias (e.g., funnel plots or statistical tests) was not performed, as no meta-analysis was conducted. Potential reporting bias is acknowledged as a limitation of the review.*
Certainty assessment	15	Describe any methods used to assess certainty (or confidence) in the body of evidence for an outcome.	*No formal certainty of evidence assessment (e.g., GRADE) was conducted. The strength of evidence was interpreted qualitatively based on consistency of findings across studies.*
**RESULTS**			
Study selection	16a	Describe the results of the search and selection process, from the number of records identified in the search to the number of studies included in the review, ideally using a flow diagram.	Results (Section 3.1 and Figure 1): 1247 records identified; 312 duplicates removed; 935 screened; 707 excluded at title/abstract; 228 full texts retrieved; 108

Section and Topic	Item #	Checklist item	Location where item is reported
			excluded; 120 included
	16b	Cite studies that might appear to meet the inclusion criteria, but which were excluded, and explain why they were excluded.	*Studies excluded at the full-text stage were categorized based on predefined criteria, including absence of pathogen-specific wastewater data, lack of public health relevance, insufficient methodological description, or lack of primary data. A full list of excluded studies is provided in Appendix A.*
Study characteristics	17	Cite each included study and present its characteristics.	Results (Tables 2 and 7): included studies cited with country, focus, pathogens, and analytical platform
Risk of bias in studies	18	Present assessments of risk of bias for each included study.	*A formal risk of bias assessment for individual studies was not conducted. Instead, methodological rigor was considered during study selection, and limitations in study quality are discussed narratively.*
Results of individual studies	19	For all outcomes, present, for each study: (a) summary statistics for each group (where appropriate) and (b) an effect estimates and its precision (e.g. confidence/credible interval), ideally using structured tables or plots.	*Individual study-level effect estimates, and statistical measures were not reported, as the review followed a qualitative synthesis approach. Results are presented in aggregated form using summary tables and*

Section and Topic	Item #	Checklist item	Location where item is reported
			narrative descriptions.
Results of syntheses	20a	For each synthesis, briefly summarise the characteristics and risk of bias among contributing studies.	*The characteristics of included studies, including study setting, pathogens, and analytical methods, are summarized in Tables 2 and 7. Formal risk of bias across studies was not assessed but is acknowledged as a limitation.*
	20b	Present results of all statistical syntheses conducted. If meta-analysis was done, present for each the summary estimate and its precision (e.g. confidence/credible interval) and measures of statistical heterogeneity. If comparing groups, describe the direction of the effect.	*No statistical meta-analysis was conducted. Results are presented through narrative synthesis due to heterogeneity in study designs and outcomes.*
	20c	Present results of all investigations of possible causes of heterogeneity among study results.	*No formal investigation of heterogeneity (e.g., subgroup analysis) was conducted. Observed differences across studies are described qualitatively.*
	20d	Present results of all sensitivity analyses conducted to assess the robustness of the synthesized results.	*Sensitivity analyses were not performed, as no quantitative synthesis was undertaken.*
Reporting biases	21	Present assessments of risk of bias due to missing results (arising from reporting biases) for each synthesis assessed.	*No formal assessment of reporting bias was conducted. This limitation is acknowledged given*

Section and Topic	Item #	Checklist item	Location where item is reported
			the narrative nature of the review.
Certainty of evidence	22	Present assessments of certainty (or confidence) in the body of evidence for each outcome assessed.	*No formal certainty of evidence assessment was performed. Findings are interpreted based on consistency and strength of reported evidence across studies.*
**DISCUSSION**			
Discussion	23a	Provide a general interpretation of the results in the context of other evidence.	Discussion (Section 4.1-4.7): results interpreted in context of global and African WBE literature
	23b	Discuss any limitations of the evidence included in the review.	Discussion (Section 4.5): limitations of WBE evidence discussed (infrastructure variability, RNA decay, quantification challenges, limited lab capacity)
	23c	Discuss any limitations of the review processes used.	*This review has few limitations. First, the absence of a formal risk of bias assessment may affect the interpretation of study quality and Second, potential publication bias may exist due to the inclusion of predominantly peer-reviewed and English/French-language studies.*

PRISMA

PRISMA 2020 Checklist

Section and Topic	Item #	Checklist item	Location where item is reported
	23d	Discuss implications of the results for practice, policy, and future research.	Discussion (Section 4.6 and 4.7) and Conclusions (Section 5): implications for early warning systems, implementation in LMICs, investment in infrastructure, capacity building
**OTHER INFORMATION**			
Registration and protocol	24a	Provide registration information for the review, including register name and registration number, or state that the review was not registered.	Methods (Section 2.1): protocol not registered in PROSPERO; narrative synthesis approach cited as rationale
	24b	Indicate where the review protocol can be accessed, or state that a protocol was not prepared.	Methods (Section 2.1): protocol was predefined but not publicly accessible; no protocol document referenced
	24c	Describe and explain any amendments to information provided at registration or in the protocol.	*No amendments to a registered protocol were made, as the review protocol was not prospectively registered.*
Support	25	Describe sources of financial or non-financial support for the review, and the role of the funders or sponsors in the review.	Funding section: "This research received no external funding"
Competing interests	26	Declare any competing interests of review authors.	Conflicts of Interest section: "The authors declare no conflicts of interest"
Availability of data, code and other materials	27	Report which of the following are publicly available and where they can be found: template data collection forms; data extracted from included studies; data used for all analyses; analytic code; any other materials used in the review.	*Data Availability Statement: no new data created; excluded studies listed in Appendix A; data extraction in Appendix B; quality assessment in Appendix C;*

PRISMA 2020 Checklist

Section and Topic	Item #	Checklist item	Location where item is reported
			included studies in Appendix D. COMMENT: Template data collection forms, analytic code, and raw extracted data are not publicly deposited in a repository.

From: Page MJ, McKenzie JE, Bossuyt PM, Boutron I, Hoffmann TC, Mulrow CD, et al. The PRISMA 2020 statement: an updated guideline for reporting systematic reviews. BMJ 2021;372:n71. doi: 10.1136/bmj.n71.
This work is licensed under CC BY 4.0. To view a copy of this license, visit https://creativecommons.org/licenses/by/4.0/
